# Women and Pensions in Italy: Gender Imbalances and the Equalization of Retirement Age

**DOI:** 10.3389/fsoc.2021.707591

**Published:** 2021-11-15

**Authors:** Nicola De Luigi, Roberto Rizza, Federica Santangelo

**Affiliations:** Department of Political and Social Science, Alma Mater Studiorum, University of Bologna, Bologna, Italy

**Keywords:** age at retirement, heckman model, gender inequalities, educational levels, pension policies, EU-labour force survey

## Abstract

This paper examines the age at retirement for men and women in Italy. Despite the expansion of women’s educational attainments, they still display lower employment rates, are frequently engaged in involuntary part-time jobs and have more fragmented careers. As a consequence, the mean age at which women receive a pension is higher than that of men. Using Labour Force Survey (2006 and 2012), the authors test the hypothesis that women’s higher age at retirement is determined by a selection bias towards more educated and work oriented women. A Heckman selection model has been developed. Results show that the main disadvantage is suffered by women with medium and low levels of education who show the highest estimated age at retirement, whereas higher educated women retire on average before men with the same level of education. The authors argue that pension policies, without interventions in the field of work-life balance policies, end up penalizing women with lower levels of education.

## Introduction

Pension reforms in nearly all industrialized countries in recent years, although principally aimed at ensuring financial sustainability, have also adopted rules aimed specifically at rebalancing gender differences, albeit in a non-uniform manner and producing outcomes that have not always been in line with expectations (Jefferson 2009; Natali and Stamati, 2013).

Pension systems are not only policy schemes with a decisive influence on intergenerational equity, but also represent intragenerational adjustment tools, especially with regard to gender relations (Casarico and Profeta, 2009).

From the early nineties, pension policies in Western countries, including Italy, introduced rules explicitly designed to counterbalance inequalities in working careers between men and women related to female disadvantages derived from discontinuous careers and prolonged periods of inactivity (Natali, 2015). These measures focused on the retirement age and the number of years of mandatory pension contributions, giving women preferential treatment. The logic followed was a ‘posteriori compensation’, offsetting the drawbacks that women accumulate during their working career, while leaving unchanged the gender imbalances relating to the division of labor within the household from which those disadvantages largely arise (Borella and Fornero, 2002).

In more recent years, however, this trend has been reversed, introducing a progressive equalization of the retirement age for men and women and, more generally, strongly limiting all the characteristics of the Italian pension system aimed at compensating the disadvantages suffered by women in the field of employment. First of all, the pension reform of 2010, the so-called Sacconi law, led to an extension to 65 years of the statutory retirement age for women in the field of public employment, in order to equate their retirement age to that of their male colleagues. Secondly, the pension reform of 2011, the so-called Fornero law, led to a gradual extension from 60 to 65 years of the retirement age of women in the private sector.

These measures evoked different opinions. On the one hand, they were welcomed because, by affirming the principle of formal equality of treatment for all contributors, they were considered an obvious measure for overcoming the patronizing idea of ‘a posteriori compensation’ for the weaker segment of the workforce. However, on the other hand, they highlighted the risk of increasing the gender gap with respect to retirement benefits, since equalization of the retirement age between men and women was not followed by any serious attempt to tackle the disadvantages suffered by women in the labor market, which are very high in Italy (Bekhouche et al., 2013).

What is more, Eurostat data from the Labour Force Survey (LFS) show that, despite the latest reforms, in the population between 50 and 69 years of age, in both 2006 and 2012, the average age at which Italian women first received an old age pension was higher than that of men.

It is such considerations that have inspired this work, which aims to investigate the retirement age of women and men^1^ in relation to their level of education.

As many scholars point out (Mäcken et al., 2021), education is a crucial variable in older workers' exit decisions, as it is linked to several individual-level factors such as workplace characteristics, income, and health. Thus, pension reforms aimed at extending working lives may affect workers differently depending on their level of education.

To shed light on the aspects highlighted, we will develop a Heckman selection model of data from the Labor Force Surveys of 2006 and 2012, which contained an ad hoc module on retirement. The aim is to observe the results of the age at retirement of men and women after correcting for self-selection and to interpret these in the light of two recent policy guidelines: the first, on a European level, focuses on the increase in female employment rates; the second, on a national level, seeks to equalize the age at retirement of men and women. Special attention will be paid to the consequences that such guidelines could have on the working career and pension of women with low human capital.

The paper is organized into five sections. The next section notes changes in Italian pension policies, paying particular attention to gender relations adjustment tools introduced recently. Section three describes recent changes in female employment rates and the relationship between such changes and education levels. Section four is dedicated to the description of data, variables and the method used for the empirical analysis. The fifth section sets out the main results. Finally, the last section proposes some considerations and conclusion.

## Women and Pension Policies in Italy

From a policy perspective, Italian pensions belong to a social insurance model in which the state provides the greater part of benefits through public earning-related schemes run on a pay-as-you-go basis. The development of the Italian pension system, notwithstanding the fact that it still absorbs more than 14 per cent of GDP (one of the highest percentages for any European country), has generated strong distortions of a distributive nature between diverse occupational categories. The very generous benefit formulas provided during the first phase of development (from World War II to around the 1980s) favored the main labor market categories (especially public employees and employees of large enterprises), whereas safeguards for the most peripheral categories (temporary and seasonal workers, the self-employed, and employees of small businesses) were much more modest.

From the mid-1970s onward, an important process of change in the pension system can be observed, in conjunction with other transformations that affected the economic-productive system, the labor market, and the demographic structure (Jessoula, 2009). From the mid-1970s and through the 1980s, Italy suffered greatly from the effects of the economic crisis following the oil shocks. These caused long-term stagflation, an increase in public debt and growing levels of unemployment arising from layoffs in large enterprises. Moreover, innovations in terms of work organization stood in contrast to the retention of older, less productive workers (Mingione and Pugliese, 2004). As a consequence, since the 1980s, measures were adopted in the form of early retirement that constituted part of a broader strategy to reduce the labor supply (“labor reduction route”), particularly of workers over the age of 50. This is a phenomenon that has, among other things, contributed to hindering the retraining and relocation of older workers, slowed the introduction of age management instruments, and more generally inhibited the implementation of active aging policies (Bertolini et al., 2016).

From the beginning of the 1990s, pension policies followed a new trend. Reforms were introduced to improve the financial sustainability and to initiate the harmonization of legislation dealing with the various professional categories (Jessoula and Hinrichs, 2012).

In this context, the retirement age for private sector employees was raised by 5 years (from 60 to 65 for men and from 55 to 60 for women) eliminating the so-called ‘baby pensions’ for public employees, along with their short contribution periods, and extending the minimum contribution period for access to old age pensions from 15 to 20 years. In addition, measures were approved that aimed to change benefits amounts, eliminate index-linked payments and extend the reference period for calculating pensionable earnings. Finally, a transition to a multi-pillar system was initiated with the introduction of the first regulatory framework for complementary ‘funded’ pensions, itself divided between Pillar II of occupational type (‘closed’ funds created through collective bargaining at a sector level) and Pillar III of ‘open’ funds managed by financial institutions such as banks, insurance companies, and financial advisors.

In subsequent years, the measures introduced were gradually strengthened through parametric adjustments designed to prolong the time spent by older workers in the labor market through an articulated series of measures that acted both on the minimum age of retirement and on benefit amounts, and a steady expansion of supplementary pensions.

In more recent years, following the recent economic and financial crisis, and as a result of international pressure on national policymaking processes, major new emergency measures were enacted in the field of social security policies. These followed the direction of increasing cuts in aiming to make cost reductions not only in the medium to long term but also in the short term.

When it comes to gender differences, pension policies have in the past recognized that women’s participation in the labor market is weaker, establishing different criteria for men and women in terms of contribution requirements. The legal retirement age of women has always been lower than men, despite their higher life expectancy. It is clear that these differences were intended to balance out the unfavorable situation of women caused by their interrupted careers and periods of unemployment not covered by social security contributions, which penalized women as far as fulfilling pension requirements was concerned.

This situation has resulted in an Italian pension system that is geared more towards the family than towards the individual. Although, formally, the unit of reference was the employee and the contribution base was earned income, pension benefits have in fact historically been required to protect the needs of the household rather than those of the individual, reflecting the historical division of roles within the household and a family organization that envisages the economic dependence of some members, such as children, the elderly and women, on others with earning capacity (Borella and Fornero, 2009).

From this perspective, specific and favorable rules for women’s pensions were introduced in order to offset gender disadvantages (Casarico and Profeta, 2009). The use of equal transformation coefficients for men and women, in the face of a higher life expectancy for women, can be regarded as an insurance premium that the system has loaded onto men, in order to finance the survivor’s pension that husbands “buy” for their wives: a kind of “couples’ insurance”, rather than individual insurance (Borella and Fornero, 2002). These reforms have taken for granted an equal distribution of income within the household (Kabeer, 2015), and have underestimated the likelihood of separation and divorce (Vignoli and Ferro, 2009). Nonetheless, women’s attitudes (Treas and Widemar, 2000) and the scarcity of pre-school services forced women to exit the labor market, at least for some years.

More recently, however, the trend has changed significantly, as previously mentioned. In a first phase, starting in 2009, the retirement age of women was changed to be the same as men in the public sector only; then the Fornero Reform extended this measure to the private sector (Bonardi, 2012). A recent report (McCracken et al., 2013) observed that the introduction of more restrictive requirements for old-age pensions and early retirement is particularly detrimental to women, whose careers are shorter and more fragmented than those of men. In fact, women spend less time in the labor market during their biographical cycle, often due to having to look after the family (Samek Lodovici et al., 2016). Therefore, stricter eligibility conditions for old-age pensions may not be met by a significant proportion of female workers, while the contribution requirements for early retirement seem to be out of reach for many women, at least in the short to medium term.

## Female Employment and Level of Education in Italy

In Italy, since the mid-seventies, female employment rates have been growing consistently, confirming a model of female participation in the labor market that is more like that of men, although at lower levels and characterized by prolonged careers.

According to Scherer and Reyneri (2008), two-thirds of the increase in female employment in Italy can be attributed to higher levels of education. The cause of this, however, is not so much determined by an increase in the employment rate of graduates or diploma holders, which has always been high, as by the relative weight, within the female population, of women graduates and diploma holders, who, even after marriage and the birth of children, are more likely to enter the labor market and stay there until retirement. Conversely, the employment rate of the poorly educated has increased very little.

Another aspect related to the increase in female employment is the spread of part-time jobs (Pissarides et al., 2005), a phenomenon that, in Italy, does not have a linear trajectory. The increase in female participation in the labor market in the seventies and eighties occurred without a parallel increase in part-time work, whereas from 1995 to 2003 approximately 40% of new jobs for women were part-time.

Many studies have highlighted the particular exposure of women to part-time jobs, but also the greater fragmentation of their professional careers due to an unequal distribution of care activities within the family, resulting in marked gender differences, both in relation to the average value of their pension income in terms of contribution years and their average age at retirement (Scherer and Reyneri, 2008). This last aspect especially sees women retire, on average, at a higher age than men. Further studies show that Italian women pay the highest penalty in Europe in terms of retirement income due to motherhood (Möhring, 2017).

The uneven growth in female employment, linked with education levels, leads us to speculate that one of the reasons why Italian women have a higher average age at retirement than men can be traced back to a positive self-selection phenomenon that push only work-oriented women to remain in the labor market. Studies highlighted that the Italian gender pay gap is one of the lowest with respect to other European countries and this is usually attributed to the Italian low level of female employment (Cutillo and Centra, 2017). Furthermore, women who enter in the labor market are usually better educated than men (Moncada, 2019; Barbieri et al., 2015). As far as occupational returns of education is concerned, Barbieri et al. (2015) argued that female prestige penalties are concentrated on low educated women and this could be due to a positive self-selection of women. Studies that take explicitly into account the self-selection in their analyses (Cutillo and Di Pietro, 2006) were mainly focused on occupational returns of the highest educated Italian workers, nonetheless they found that women are less likely to be overeducated then men. Authors explained this phenomenon with “a lower pressure to work among female in a traditional society as the Italian one” (*p*. 158). The expectation, therefore, is that by correcting the analysis to take account of such self-selection, the differences detected also in age at retirement will be mitigated. The high incidence of part-time work, by contrast, could be one of the reasons why women with low levels of qualification postpone their retirement longer than men with similar educational qualifications.

Further study into the relationship between retirement age and the level of education has two research objectives. We know that retirement age increases in proportion to the level of education (Hofäcker et al., 2015): on the one hand, staying in education delays the start of employment; on the other, high levels of education are generally associated with better jobs, thus motivating employees to remain in the labor market for as long as possible. In this regard, one of the assumptions of this research is that the retirement ages of men and women differ only among those who have low levels of education. With regard to the less educated, it is in fact reasonable to expect the retirement age for women to be higher than that of men, due of their higher number of career breaks. The second research objective concerns the specific composition of employed women in Italy. Scherer and Reyneri (2008) have shown that the increase in employment levels of women has been caused largely by the structural change in the composition of the female workforce in terms of level of education, following a considerable increase in the number of female diploma holders and university graduates. The assumption is that the average age at retirement for women is higher than that of men because there is a strong imbalance of employed women towards those with high levels of education and/or those with a greater attachment to work.

## Data and Methods

Analysis was performed on the two European Labour Force Surveys (EU-LFS) (2006 and 2012), which contain an ad hoc module on retirement (“Transition from work into retirement”). Despite some limitations, which will be set out below, the adoption of this specific database is not unusual in the study of retirement behaviour^2^ (Radl, 2013). It will allow future comparisons with non-European countries that are not covered and not comparable by other datasets like SHARE. Furthermore, Italian statistics on pension age in the OECD datasets use exactly the European Labour Force Surveys. EU-LFS are developed quarterly on 35 countries and it is the largest European household sample survey, whose main objective is to classify the population of working age^3^. Our analysis focuses on retirement age: for this reason, it was necessary to take into consideration a sub-sample, consisting of subjects between 65 and 69 years of age. The in-depth section on retirement was intended for interviewees aged between 50 and 69 years who had worked until at least the age of 50 (Table 1). The decision to further reduce the age range was determined by methodological considerations: among individuals between 50 and 64 years of age, the percentage of employment is still very high; therefore, focusing only on those who have exited the labour market and have thus reached retirement age would have significantly distorted the analysis.

**TABLE 1:** Description of the dataset and sub-sample of 65–69 year olds.

	65–69 year olds	Not employed at 50 years of age (A)^a^	Employed at 50 years of age^b^		Sub-sample (A + B)
			Pensioners (B)	Working (employed or seeking work)	
2006	10,981	3,388	5,744	721	9,132
2012	9,159	2,816	5,136	685	7,952
Total	20,140	6,204	10,880	1,406	17,084

a Individuals 65–69 year old that are in the dataset but not entitled to answer to ad hoc section on retirement because they left (or never entered) the labor market before 50 years of age.

b Individuals 65–69 year old entitled to enter the ad hoc section of the questionnaire on retirement because they were employed at least up to 50 years of age.

In the older age group, however, more than nine people out of 10 have already retired; we expect, therefore, a better balance between those who have accrued the necessary contribution years to retire and those who have reached the maximum age limit.

The sub-sample of retirees used is therefore composed of individuals who receive a pension, who call themselves pensioners, who had exited the labour market prior to the interview and who did not begin receiving a pension before the age of 50.

The dependent variable is the age at which a person first receives a pension. In the literature, there is not a clear definition of the concept of pensioner; nevertheless, it is customary to associate an exit from the labour market with respondents who perceive themselves to be pensioners (Denton and Spencer 2009). The available database, however, only provides the ages of individuals in 5-year bands. It would, therefore, have been very inaccurate to use this criterion, given it would not be possible to define accurately the age at which the interviewees left their last paid job. For this reason, we chose to associate the self-perception of being retired with the age at which a pension is received, while ensuring that the interviewee had actually exited the labour market and had not received a pension before the age of 50 (Damman et al., 2015).

Before turning to the description of the variables and the technique used, we need to further clarify the methodology: the data examined were not longitudinal, and therefore do not allow us to correlate the time of receipt of a pension with the meeting of the requirements and with the eventual choice to remain, nonetheless, in the labour market. Therefore, any empirical evidence that is encountered will not be interpreted in causal terms, but will only indicate the existence of a relationship between certain variables and the retirement age.

The variables whose covariation with age at retirement we intend to investigate are, among socio-biographical variables, gender and level of education. The latter was recoded following the isced classification into three modalities: 0–2 low, 3–4 medium and 5–6 high. Note that, for those who are retired, information on the last job appears only for those who left the labour market in the 9 years preceding the interview. Therefore, taking the last job into account would have dramatically reduced the sample.

The geographical area of residence and year of the survey are control variables, organised in two modalities (centre-north, south and islands; 2006 and 2012 respectively). A final relevant control variable relates to the presence of a cohabiting partner (Blau and Riphahn, 1999) who might affect a coordinated decision of retirement within the couple.

Many scholars who have studied gender differences in retirement age have highlighted the importance of taking into account the different rates of male and female activity and employment. Despite the fact that legislation had, up until a few years ago, protected women, discrepancies (if any) could be traced back to the self-selection of the female sample, composed of subjects with a particular propensity to work (Radl, 2013), especially in countries where the percentage of working women is low. The use of the Heckman selection model (1979) with maximum likelihood estimates corrects the distortion of the sample (Hess, 2017). The procedure consists of two equations. The first is the selection model, a *probit* equation that estimates the probability of being employed at the age of 50, distinguishing between those who are retired and those who are not eligible for retirement (the disabled, housewives, and those not in employment)^4^. The second is a linear regression of age at retirement.

When estimating the selection equation, we need to introduce variables defined as instrumental. For this purpose, we introduced marital status, taking into account the interaction with gender. Married men, in fact, have a greater chance of being employed than men without their own family unit. For women, on the other hand, the probability of being employed is greater if they are not married (Lucchini eta al., 2007). An important control variable used is the presence of children. It is common knowledge that children influence the working choices of women, who tend to abandon the labour market when their first or, failing that, their second child is born. In both cases, the dataset provided by Eurostat offers partial information: both marital status and the presence of children living at home describe the situation of individuals at the time of the interview, which therefore cannot be correlated with the past, that is to say the moment when - for women typically in middle age groups - the decision to abandon the labour market was taken^5^. Nevertheless, as we shall see in the next paragraph, both variables show what we would expect. The area of residence and year of interview are the same control variables we also introduced in the *probit* equation^6^.

Using the Heckman procedure, from the *probit* equation on the probability of being employed, we estimate the Inverse Mill’s ratio which, if statistically significant, confirms the presence of self-selection of the sample and is used in the second equation to correct the estimates obtained by linear regression.

## Results

### Descriptive Findings

Table 2 shows the average age at retirement, according to levels of education. At first, it appears that the average age at retirement of women increased between 2006 and 2012, while that of men decreased. This trend results in women entering retirement, on average, after their male colleagues in2012^7^. But a drill-down to analyze the data according to education level reveals some interesting peculiarities. Male and female university graduates, in fact, did not undergo any noteworthy changes between 2006 and 2012 and a higher average age at retirement was recorded among male graduates in both years. The decrease among men is attributable to the slight decrease in the average age at retirement of subjects without any diplomas.

**TABLE 2:** Average pension age (and *standard deviation*) by educational attainment and sex.

Level of qualification	Low	Medium	High	Total	Low	Medium	High	Total
	2006				2012			
Men	58.8	59.0	60.5	58.9	58.4	59.0	60.9	58.7
*Standard Deviation*	*4.41*	*4.21*	*3.85*	*4.37*	*4.53*	*4.30*	*4.12*	*4.49*
N	2,899	583	181	3,663	2,294	784	203	3,281
Women	57.7	58.6	59.0	57.9	59.1	59.6	59.0	59.2
*Standard Deviation*	*3.45*	*3.85*	*3.65*	*3.53*	*3.38*	*3.62*	*3.93*	*3.49*
N	1,671	294	116	2,081	1,296	364	195	1,855

The relationship between levels of education and retirement age assumes different characteristics in relation to gender. In the case of men, in 2012 we noted that an increase in the level of education also increases the age at which a pension is first received. The reasons are numerous and have been widely discussed in the previous paragraphs.

For women in 2006, the age at retirement of those with low levels of education is lower than that of women with medium or high levels of education, while in 2012 the situation is more complex: women holding a medium level of education have a higher average age at retirement than women with low and high levels of education. Between 2006 and 2012, the greatest increase in terms of age at retirement was related, however, to both women with low and medium levels of education. In 2012 these two social categories retire, on average, after their male colleagues who have the same qualifications. Once again, the exception is high educated, who, on average, receive their pension before their male colleagues (up to around 2 years prior for retirees interviewed in 2012).

### Selection Bias

The data thus presented reveals some irregularities. However, it is acceptable to assume that a positive selection mechanism is in place, whereby women who remain in employment up to the age of pension entitlement are a very special social category, characterized by a particularly pronounced attachment to work. The hypothesis that pensioners in the 65–69 year-old age range who receive a pension are selected rather than those who are not entitled to a pension is indeed confirmed in Table 3 by the statistical significance of the *inverse Mill’s ratio*. In fact, the *reverse Mill’s negative ratio* shows that, in the absence of a correction factor, we would have underestimated the average age at retirement.

**TABLE 3:** Heckman selection model: *probit* on the probability of being employed until the age of 50 and linear regression on the age at retirement.

	Mod. 1	Mod. 2
	Age of retirement	Probability being employed
Sex (ref. men)		
Women	1.32^***^	−1.27***
Residence (ref. Centre/north)		
south and islands	2.35^***^	−0.25***
Educational attainment/education (ref. medium)		
Low	−0.16	−0.22***
High	1.58^***^	−0.12
Year of survey (ref. 2006)		
2012	0.33^°^	−0.10***
Year of surv. #educational attainment (ref. 2012#medium)		
2012#low	0.13	
2012#high	0.16	
Educational attainment/education (ref. medium#female)		
low#female	0.61^***^	−0.19***
high#female	−3.63^***^	0.90***
Presence of partner (ref. absent)		
Respondent has a partner	−1.17^ ***^	
Sex#presence of partner		
female# has a partner	1.58^***^	
Year of surv.#sex (ref. 2006#female)		
2012#female		0.20***
Marital status (ref. widowed, separated, divorced)		
Single		−0.29***
Married		0.17***
Sex#marital status (ref. woman#wid. div. sep)		
female#single		0.55***
female#married		−0.31**
Number of children (ref. 0)		
1	0.09**
2+		−0.03
No. children#sex (ref. 0#female)		
female#1		−0.10**
female#2+		−0.15*
Constant	59.61^***^	1.52***
Inverse Mill’s ratio	−4.26***	
Wald chi2 (11)	1501.48^***^	
N	10,880	17,084

Proceeding by steps, the first equation on the probability of being employed at age 50 produced estimates fully in line with expectations: being a married woman with at least one child reduces the probability of remaining in the labor market up to 50 years of age, whereas being single increases it. Men, on the contrary, are more likely to be in work if married, and the presence of children does not seem to influence their level of employment. Not having one’s own family increases the probability of being unemployed or of having career breaks prior to fifty years of age. For both men and women, being widowed, separated or divorced places them in an intermediate position: the likelihood of working is greater with reference to married women, and lower with reference to married men.

Over time, male employment rates have slightly reduced, but, as noted, this does not apply to women, whose employment rates are instead increasing. In fact, between 2006 and 2012, the probability of women having a job that entitles them to a pension increases. Considering that, in the 2006 data, we are observing women born between 1937 and 1941 and, in the 2012 data, those born between 1941 and 1947, data from the selection equation show a higher propensity for female employment in the younger cohorts.

The level of education correlates with participation in the labor market: the higher the cultural capital, the higher the probability of being employed and receiving an old-age pension. Women with a higher education have greater probability than man of being employed and of receiving an old-age pension. Moreover, from a geographical point of view, Italy is divided into two: the probability of being employed until at least fifty years of age is greater among those living in the center and north than in the south.

In summary, the selection equation results confirm what the literature already tells us: that female employment rates, while increasing over time and by birth cohorts, remain far below those of men and the probability of remaining in the labor market differs according to geographical area, level of education and marital status. The pensioner population is therefore, as expected, highly self-selected. Estimates of the second retirement age equation, when corrected for that distortion, provide, in other words, a framework of variables correlated to retirement ages, cleansed of this selection effect, which hides, obviously, other individual unobservable characteristics for which the database does not provide information, in terms of attachment to work, motivation, and so on.

### Age at Retirement and Gender Differences

**FIGURE 1:**
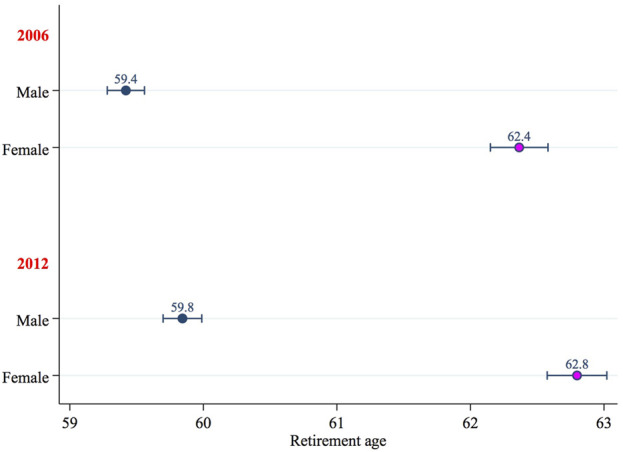
Estimated age at retirement by sex and year: Predicted values and 95% confidence intervals.

According to the main equation of the Heckman model, all other characteristics being equal, women, on average, retire about 3 years later than men (Figure 1). According to Radl (2013), something similar takes place in only four other countries: Spain, Finland, Ireland and France, where, in most cases, women’s employment rates are lower than those of men^8^.

We know, however, that female employment rates are highly influenced by the education levels. While it is true that the cost of leaving the labor market because of family responsibilities increases for women graduates, it is also true that their bargaining capacity within the couple increases (Brines, 1993; Lucchini et al., 2007). It follows that the female sample of retirees is strongly selected in favor of women with high levels of education.

**FIGURE 2:**
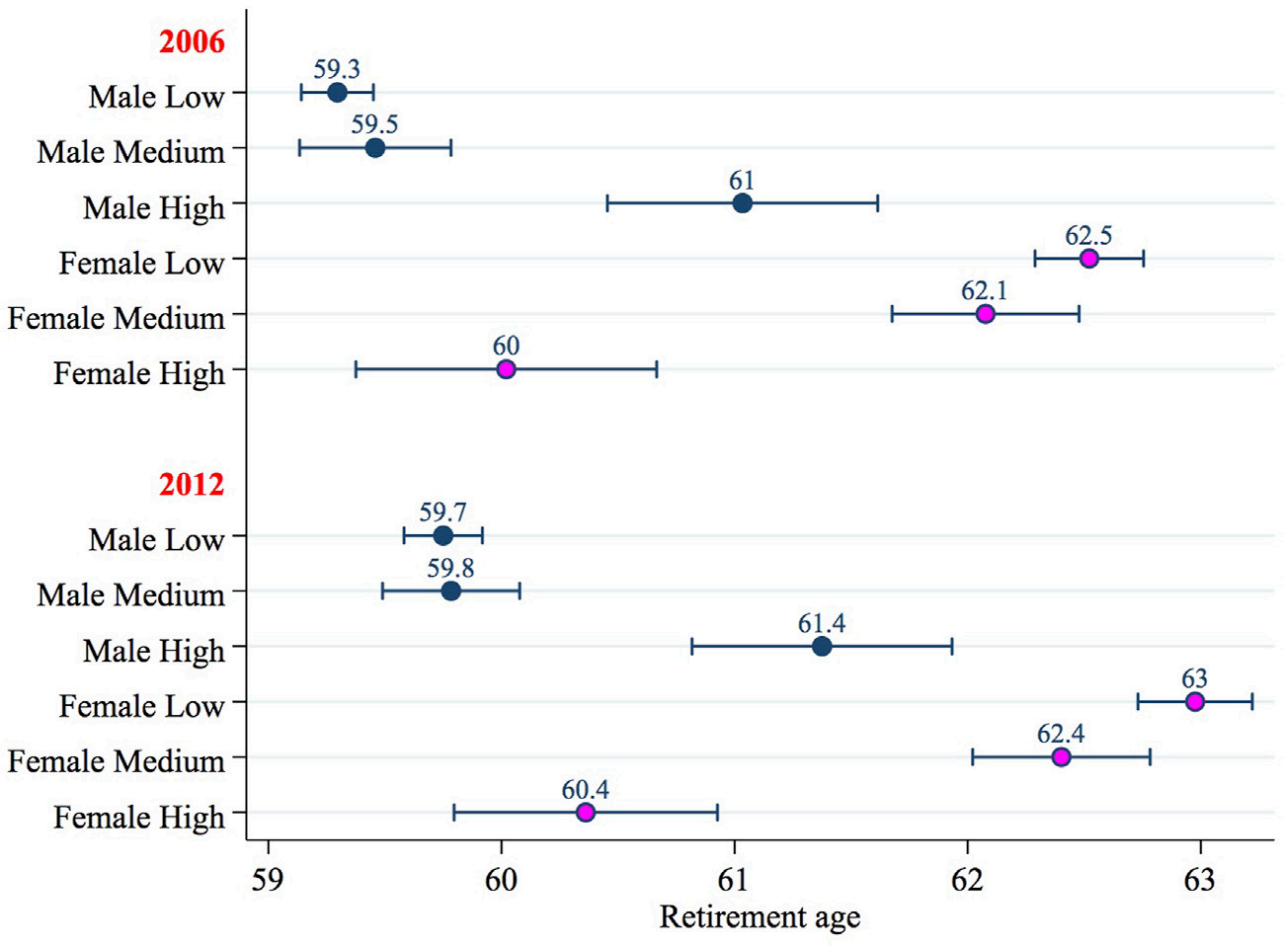
Estimated age at retirement by sex, year and educational attainment: Predicted values and 95% confidence intervals.

Figure 2 illustrates even more concisely the predicted age at retirement by gender and level of education according to the year of the survey. The estimate is calculated by averaging every other variable in the regression. The predicted values were calculated considering men and women as though they were two separate samples.

For men, the age at retirement increases in line with the attainment of higher levels of education, while for women precisely the opposite is true. Men who have a degree or a high school diploma tend to remain in employment until reaching the maximum retirement age. People with low levels of education, however, are likely to carry out less rewarding and often more arduous work. Accordingly, having attained the number of contributory years required to accrue a pension is often a sufficient reason for retirement.

Conversely, almost only one out of five women (compared with one out of two men)^9^ manage to retire after meeting the contributory requirements, and among those with low levels of education, the high instability of their working careers requires that they prolong their number of working years compared with their male colleagues. Consequently, this appears to confirm the results that have emerged in other research: fragmented working careers, widespread part-time work, and the income gap between men and women for the same work all mean smaller pension *cheques* and longer careers for women than for men.

Female graduates, however, who retire almost a year before their male colleagues, can afford to exit the labor market at a younger age than women with lower qualifications (Figure 2). In fact, they usually have access to better protected jobs, for example in the public sector, and can more frequently negotiate flexible working hours at times when they need to tend to the family or children, instead of being forced to leave the labor market.

Turning to the comparison between 2006 and 2012, in the period observed, the age at retirement increased and the pre-existing gender differences were exacerbated to the detriment of women with low levels of education. In fact, gender equality in terms of retirement age, provided by the pension reforms, which will be visible in the next pensioner cohorts, if not accompanied by labor policies aimed at reducing gender differences (such as in income, access to management positions, distribution of part-time work, the burden of family duties, etc.), will only increase the existing gap in retirement age between men and women, to the detriment of women employed in low-skilled occupations in particular.

## Conclusion

The many studies that have addressed the relationship between women and the labor market in Italy have focused on gender inequalities such as the lower rates of activity and employment of women, the inequality in the salaries men and women receive, and the difficulties faced by women in terms of work-life balance.

However, little has been done to investigate the gender differences relating to the conclusion of working life and retirement age. In most industrialized countries, women retire, on average, at a younger age than men (König, 2017). In Italy, however, women gain a pension, on average, after men. This phenomenon is related to two main factors: the self-selection of women reaching retirement and the fragmentation of women’s working careers.

It was thought that, by raising the retirement age, recent reforms would increase people’s age at retirement, involuntarily in the case of individuals with low levels of education, voluntarily in the case of those who are in well-remunerated and prestigious occupations. The data does not allow us to assess the impact of these reforms, whose effects will only begin to be evident in the coming years. However, the analysis has shown that the self-selection of women with high levels of education hides the big disadvantage faced by women with low and medium levels of education. In fact, if these women remained in the labor market until the age of fifty, there would be an increase of almost 3 years of the age at which they receive a pension, higher than that of men in the same situation. Furthermore, our results show that statistics on average age at retirement by sex must be considered carefully because of the selection bias of high educated women, particularly in countries with low level of female employment rates. Low educated women, in fact, are more prone to be inactive before the retirement age but when employed they are forced to cope to more uneven financial retirement options than their male counterparts. On the contrary, highly educated women – who are highly present among retired, and strongly affect statistics on retirement by sex – prefer to exit before men.

Investment in education has an undoubted advantage for female university graduates in terms of age at pension, since it is lower than for women with low levels of qualification and for men with the same qualifications. The first of the two factors considered (the self-selection of employed women) allows us, then, to grasp gender inequalities in terms of even higher ages at pension.

In general terms, we can also say that the pension age of women is higher than that of men with the same levels of education because of their shorter career path, their more frequent breaks due to care obligations, their higher number of part-time contracts and the reduced pay received by them for the same work. These elements end up having the effect of postponing the pension age due to lack of eligibility requirements, or because it is economically disadvantageous. The second factor (fragmented women’s careers) considered in our assumptions therefore goes in the direction we would expect.

In conclusion, a further remark appears relevant. Some research has underlined the importance–and the widespread practice in Italy–of grandparents caring for their grandchildren, describing a potentially undesirable and unexpected effect on female employment of pension reforms aimed at increasing the retirement age (Bratti et al., 2016). The presence of grandparents–especially of grandmothers–who are eligible for retirement (Hank and Buber, 2009), increases the chances that daughters or daughters-in-law have to find or keep a job. The absence of grandparents willing to spend their own free time looking after their grandchildren, because they are still in employment, could therefore result in a reduction in female employment.

The contribution of this article is a further step towards understanding gender inequalities: pension policies aimed at pushing up the retirement age of women could further widen the gap between men and women, penalizing especially those who hold low or medium levels of qualification. Considering that female employment tends to consist primarily of highly educated women, the only way of getting close to the objective, defined by international institutions and widely accepted by the scientific community, of increasing women’s participation in the labor market, is by promoting the employment of the less educated. The issue is that these women would persistently suffer from the social costs caused by fragmented and longer careers, due to the difficulty they have in achieving the eligibility criteria for retirement, on the one hand, and the postponement of the retirement age envisaged by the most recent guidelines, on the other.

Given this, the blanket affirmation of a principle of formal equality of treatment for all contributors and overcoming the paternalistic idea of ‘a posteriori compensation’ would conceal the perpetuation of gender inequalities produced by working careers. Therefore, pension measures that are not properly linked to policies aimed at mitigating the gender gap in the labor market would appear to contradict the aim of achieving equality (Heckman, 1979; Becker, 1996; Del Boca et al., 2005).

## Data Availability

Publicly available datasets were analyzed in this study. This data can be found here: https://ec.europa.eu/eurostat/web/microdata/european-union-labour-force-survey.

## Footnotes

1 Eurostat main indicator (average exit age from the labour force) is estimated with a probabilistic model based on LFS, but there “is no internationally harmonized statistical definition of retirement” (http://ec.europa.eu/eurostat/cache/metadata/en/lfsi_exi_a_esms.htm). In this paper we define age at retirement as the age at which a person (who self-perceives retired) first received a pension and is actually inactive.

2 This paper originates from the author’s participation in the research project “Determinants of Retirement Decisions in Europe and the United States: A Cross-National Comparison of Institutional, Firm-level and Individual Factors ”, coordinated by Dirk Hofäcker and by Mannheimer Zentrum für Europäische Sozialforschung (Hofäcker et al., 2016).

3 For further details visit: https://ec.europa.eu/eurostat/web/lfs.

4 Not having been employed until at least the age of 50 is, in fact, a proxy for exiting from - or a failure to enter - the labour market and, in fact, in 48 per cent of cases involves people–98% of whom are women - who have never worked, while the remaining percentage abandoned the labour market at 33–37 years of age on average.

5 The variables that include children at home were constructed only in respect of the interviewee: the children of partners, if not their own, were not included. It is plausible to imagine that extended families were formed subsequent to the time when the decision to leave the labour market or remain there was taken.

6 We control the assumption of normal form of covariates in the *probit* model.

7 On the sample of 50–69 year olds, in 2006 too, women’s average retirement age is higher than that of men, but restricting the sample to the percentage of older people does not allow for insightful analysis.

8 Please note that we are considering the age at which one has received a pension, not the age at which one has exited the labour market, which in Italy is higher for men than for women (Oecd, 2017).

9 In 2012 respondents were asked on reasons for having left the labor market.
