# Supplementary material for: Women and Pensions in Italy: Gender Imbalances and the Equalization of Retirement Age
Source: Front Sociol. 2021 Nov 15;6:707591. doi: 10.3389/fsoc.2021.707591 (PMC8634470; doi:10.3389/fsoc.2021.707591)
Supplement: Supplementary file 1 [file Table1.DOCX]

**Supplementary Materials**

Figure 1 Predicted values of age at retirement by sex, 95% confidence intervals.

Data for figure 1

| Year | Sex | age at retirement | 95% confidence interval | |
| --- | --- | --- | --- | --- |
| 2006 | Male | 59.4 | 59.3 | 59.6 |
| 2006 | Female | 62.4 | 62.1 | 62.6 |
| 2012 | Male | 59.8 | 59.7 | 60.0 |
| 2012 | Female | 62.8 | 62.6 | 63.0 |

Figure 2 Predicted values of age at retirement by sex and level of qualification, 95% confidence intervals.

Data for figure 2.

| Year | Sex | Level of education | age at retirement | 95% confidence interval | |
| --- | --- | --- | --- | --- | --- |
| 2006 | Male | low | 59.3 | 59.1 | 59.5 |
| 2006 | Male | Medium | 59.5 | 59.1 | 59.8 |
| 2006 | Male | High | 61.0 | 60.5 | 61.6 |
| 2006 | Female | low | 62.5 | 62.3 | 62.8 |
| 2006 | Female | Medium | 62.1 | 61.7 | 62.5 |
| 2006 | Female | High | 60.0 | 59.4 | 60.7 |
| 2012 | Male | low | 59.7 | 59.6 | 59.9 |
| 2012 | Male | Medium | 59.8 | 59.5 | 60.1 |
| 2012 | Male | High | 61.4 | 60.8 | 61.9 |
| 2012 | Female | low | 63.0 | 62.7 | 63.2 |
| 2012 | Female | Medium | 62.4 | 62.0 | 62.8 |
| 2012 | Female | High | 60.4 | 59.8 | 60.9 |
